# Impact of Polyvalent Mechanical Bacterial Lysate on lymphocyte number and activity in asthmatic children: a randomized controlled trial

**DOI:** 10.1186/s13223-020-00503-4

**Published:** 2021-01-20

**Authors:** Małgorzata Bartkowiak-Emeryk, Andrzej Emeryk, Jacek Roliński, Ewelina Wawryk-Gawda, Ewa Markut-Miotła

**Affiliations:** 1 Department of Clinical Immunology, Medical University of Lublin, Lublin, Poland; 2 Department of Paediatric Pulmonology and Rheumatology, Medical University of Lublin, Lublin, Poland

**Keywords:** Bacterial lysates, Allergic asthma, Lymphocyte, Immune, Children, Patients, Infection

## Abstract

**Background:**

Polyvalent Mechanical Bacterial Lysate (PMBL®) contains antigens of bacteria responsible for respiratory infections. PMBL® has been proven to reduce the number of respiratory infections, and in its use, immunological benefits have been seen in allergic patients. PMBL® activates both innate and specific immune responses. The lysate induces dendritic cells, T and B lymphocytes and IgA secretion, as well as the production of antibodies directed against administered bacterial antigens. Moreover, it increases the response against other bacteria and viruses. The immunologic mechanism of lysate’s action is not yet clearly determined. The objective of this study was to assess the effect of PMBL® on T cells in children with allergic asthma.

**Methods:**

This study was a part of the EOLIA study. Herein, 49 children with allergic asthma and house dust mites allergy were included: 21 in PMBL® and 28 in the Placebo group, both, drug and placebo were administered sublingually. The tests were done at baseline and 12 weeks after the last tablet intake. The lymphocytes CD45+, lymphocytes T CD3+, CD3+CD25+, CD3+CD69+, Th CD3+CD4+, CD4+CD25+, CD4+CD25+ high, CD4+CD69+, Treg CD4+CD25+FOXP3, Tc CD3+CD8+, CD8+CD25+, CD8+CD69+, NK-like T CD3+CD16+CD56+ and NK cells CD3−CD16+CD56+ were described.

**Results:**

At baseline, no significant differences between groups relative to blood count cells were observed, except for eosinophils. After 12 weeks, we observed an increase of T lymphocytes count. In addition, CD4+CD25+FOXP3+, CD8+ and CD3−CD16+CD56+ and (insignificantly) Th count increased. However, CD69+ and CD25+ subset of CD3+ significantly decreased.

**Conclusions:**

The EOLIA study demonstrated that PMBL® administration 10 days per month for 3 months changed the panel of T lymphocytes.

*Trial registration* Clinical Trial Registration: This study was a part of the EOLIA (Efficacy Of mechanical bacterial Lysate In Allergic children), a clinical study NCT02541331. Frederic Durmont, MD Lallemand Pharma International AG. Date of registration 09/08/2013. URL of trial registry record: https://clinicaltrials.gov/ct2/show/study/NCT02541331.

## Background

PMBL® is a lyophilized Polyvalent Mechanical Bacterial Lysate and is obtained by mechanical lysis of 48 billion bacteria commonly responsible for upper and lower respiratory infections (*S. aureus*, *S. pyogenes*, *S. oralis*,* K. ozaenae*, *H. influenzae*, *K. pneumoniae*, *M. catarrhalis *and *S. pneumoniae*) [1].

In clinical trials, PMBL has been shown to reduce the number of respiratory infections in patients suffering from recurrent respiratory infections [2, 3]. Upon administration, the Cochrane systematic review and meta-analysis (2006, updated 2011) assessing bacterial lysates concluded that the frequency of acute respiratory tract infections decreased by 40% [4, 5].

PMBL induces a specific immune-stimulation against the 13 bacterial strains, activates both innate and specific immune responses and has the capacity of inducing maturation of dendritic cells [1]. This initial stimulation of innate immune system is brought about by the recruitment of a functionally efficient population of T and B lymphocytes, and results in the secretion of specific IgA and production of opsonizing antibodies directed against the administered bacterial antigens [6, 7]. In addition, the demonstration of a polyclonal activity of PMBL seems to be important in suggesting that an efficient immune response could be raised by treatment, not only towards pathogens present in PMBL, but also to other bacteria and viruses responsible for respiratory infections [5, 8].

More recently, possible benefits of bacterial immune-stimulation have been suggested in allergic patients suffering from allergic rhinitis [9, 10], asthma [11, 12] or atopic dermatitis [13]. In children with established asthma, respiratory tract infections play a key role in triggering attacks of wheezing and acute exacerbations. Furthermore, allergy and viral infections synergistically increase the risk of acute exacerbation [14–17].

The impact on T lymphocytes of sublingual bacterial lysates is not still clearly determined. The current data suggest that the use of PMBL leads to CD4+ and CD8+ T cell activation and increase of cytokines such as INF-γ secretion in vitro [5, 18]. However, these studies are scarce and do not concern asthmatic patients.

The objective of this study was to demonstrate specific changes in a panel of cellular immunological response parameters after treatment of PMBL® in children with allergic asthma.

## Methods

This study was a part of the EOLIA (Efficacy Of mechanical bacterial Lysate In Allergic children, NCT02541331), a clinical study on the clinical course of asthma and related immunological parameters in asthmatic children. This was a randomized, double blind, placebo-controlled multicentre, parallel-group study, and was conducted in Poland between July 2014 and June 2015 [12].

The study protocol was approved by the Bioethics Committee of the Medical University of Lublin (Bioethic Committee resolution number KE-0254/140/2014) and was conducted according to the Declaration of Helsinki. Written informed consent was obtained from parents of patients before enrollment in the study, and a signed assent was obtained from all patients.

The population of the study was paediatric (6 to 15 years) with allergic asthma and with house dust mites (HDM) allergy confirmed by positive skin prick test (Allergopharma-Nexter Sp. z o.o.—Poland) or elevated serum anti-IgE specific level with HDM allergens (Polycheck®, Biocheck-GmbH, EMMA-MDT Sp. z o.o.—Poland). In the biology subset, 49 patients of the Children’s Hospital of Lublin were included, 21 and 28 patients in the PMBL® and in the placebo groups, respectively. HDM allergy in this subset was confirmed with positive Skin Prick Tests (SPT) in 42 children, elevated serum specific IgE level in 4 children or with both methods in 3 children.

PMBL® Tablet (Ismigen®, Lallemand Pharma AG, Massagno, Switzerland) contains 7 mg of bacterial lysate from the following bacteria: *Staphylococcus aureus*, *Streptococcus pyogenes*, *Streptococcus *(*viridans*)* oralis*, *Klebsiella pneumoniae*, *Klebsiella ozaenae*, *Haemophilus influenzae Serotype B*,* Neisseria catarrhalis*, and* Streptococcus (Diplococcus) pneumoniae* (six strains). Patients received sublingually one tablet per day before breaking fast, on the first 10 days of each month, for three consecutive months. Drugs were provided by the sponsor, it was the same product as is commercially available on the market. Tablets were administrated by parents at home.

Main inclusion criterion were: allergic asthma diagnosis according to GINA guidelines with house dust mites (HDM) allergy prior to screening [19, 20]; partly controlled or uncontrolled asthma prior to screening visit; treatment with inhaled corticosteroids (ICS) and short acting beta agonist (SABA) used against dyspnea or ICS with long acting beta agonist (LABA) during the previous three months; and at least two exacerbations of asthma within the 12-month period before study.

Exacerbations were assessed and validated by the investigator on the basis of deterioration in symptoms and temporary change in treatment. Severity of asthma exacerbation was defined as:

1. Mild/moderate if requiring a transient increase in ICS/β2-ago-nists/anticholinergic use for ≥ 2 days, or an emergency room visit but without prescription of systemic corticosteroids.
2. Severe if requiring hospitalization or emergency room visit and systemic corticosteroids to be prescribed or systemic corticosteroids (oral or parenteral) to be prescribed for ≥ 3 days but < 7 days.

All the inclusion and non-inclusion criteria were published recently [12].

All children were treated for asthma with constant doses of ICS or ICS with LABA and SABA as needed for the entire study period [20]. Patients administered bacterial lysate immunostimulation and/or allergen-immunotherapy within the previous 6 months before screening were excluded. By extension, flu vaccination, chronic pharmacotherapy with systematic corticosteroids or leukotriene receptor antagonists was not permitted during the treatment period.

According to the randomisation list, the treatment numbers were allocated to either PMBL® or Placebo. The biology subset was done before treatment start (baseline), and 12 weeks after the last period of tablet intake.

### Immunological parameters

At inclusion and 12 weeks after the last tablet intake, complete blood count was evaluated. This included red blood cells, white blood cells (neutrophils, basophils, eosinophils, lymphocytes, monocytes) and platelets count. The following lymphocyte phenotypes were described (count and % of lymphocyte):

- The lymphocytes CD45+ (the leukocyte common antigen, CD45+),
- T lymphocytes (CD3+), late activated T lymphocyte (CD3+CD25+), early activated T lymphocyte (CD3+CD69+),
- T helper lymphocytes (CD3+CD4+), late activated T helper lymphocytes (CD4+CD25+, CD4+CD25+ high), early activated T helper lymphocytes (CD4+CD69+),
- T reg lymphocytes (CD4+CD25+FOXP3),
- T cytotoxic lymphocytes (CD3+CD8+), late activated T cytotoxic lymphocytes (CD8+CD25+), early activated T cytotoxic lymphocytes (CD8+CD69+),
- NK-like T lymphocytes (CD3+CD56+CD16+),
- NK cells (CD3−CD16+CD56+).

### Flow cytometric analysis of phenotype of T, B, and NK cells at baseline and after 12 weeks follow up

Determination of absolute counts of lymphocytes subpopulations was carried out by flow cytometry software based on differential expression of CD45+ and other surface antigens on study cells. A flow cytometer was used to determine the cells of interest as a percentage of a ‘reference’ population. A haematology analyser then provided an absolute lymphocytes number which allowed the determination of the absolute number of lymphocytes CD45+.

The peripheral blood mononuclear cells (PBMCs) were collected with Pasteur pipettes and washed twice with Ca2+Mg2+-free PBS for 5 min. Following density gradient centrifugation, the PBMCs underwent two-color or three-color labeling with adequate quantities of monoclonal antibodies, according to manufacturer’s instructions. The isolated suspension of cells was divided into individual test tubes at 1 × 10^6^/sample and incubated with monoclonal antibodies. 20 μL of antibodies was added to each sample of evaluated cells and incubated at room temperature for 20 min. Following incubation, the cells were washed twice with PBS (at 700 g, 5 min) and immediately analyzed with the FACS Canto II flow cytometer (Becton Dickinson, US), equipped with two lasers of 488 nm and 633-nm wavelength. Data acquisition was conducted with specialist FACS Diva Software 6.1.3 (Becton Dickinson, US). The data were analyzed with CellQuest Pro (Becton Dickinson, US). CaliBRITE (Becton Dickinson, US) was the calibrating system used to optimize flow cytometer settings. BD FACSDiva software collected data from each compensation control tube and automatically calculates accurate compensation values for each fluorochrome combination. Then, the median fluorescence intensities (MFIs) of the positive and negative populations of the compensation control were aligned in the neighboring channels. For each sample, data was acquired by routine collection of 30,000 events in the lymphocyte gate (region R1) in a forward-scatter (FSc)/side-scatter (SSc) dot plot. Cells populations were determined by differences in cell size and granularity. This allowed for erythrocytes, platelets, dead cells, and cell fragments to be excluded from analysis. Labeled cells were recorded based on the created lymphocyte which gate purity was evaluated on the basis of cell distribution relative to the CD45 FITC/CD14 PE coordinate system. The proportion of positively labeled cells was assessed. For a positive result we were looking for the shift in intensity between negative control and a positive samples. Flow cytometry results were presented as the proportion of cells stained with monoclonal antibodies conjugated with fluorescent markers, or fluorochromes. The antibodies anti CD3+, CD4+, CD8+, CD25+, CD69+, FOXP3+ conjugated with their appropriate fluorochromes FITC, PE, PerCP, PerCP-Cy™5.5, PE-Cy™7, APC, APC-Cy7 were used.

### Statistical analyses

All data were entered in the database for recording purpose only. The change from baseline of each laboratory parameter was described and compared by within- and between-group tests, using Student t-test. In case of non-normality (assessed by the Shapiro–Wilk’s test), Wilcoxon’s test was applied. The statistical analyses were performed using the SAS software (Version 9.4 for Windows). A *p*-value < 0.05 was considered as statistically significant.

## Results

The EOLIA study was conducted in four cities in Poland in the years 2014–2015. A total of 152 patients were randomized in the study, five patients did not complete the study.

We randomly enroled 60 patients to the biology subset. This sample size was calculated using the power test procedure and sample size test in Statistica Statsoft Software based on previous studies concern effects of immunostimulants on CD3+ lymphocyte count [5, 21]. A total of 22 evaluable patients per group were to be enrolled to ensure a power of 90% to detect the abovementioned group difference, with an α level of 0.05 and standard deviation of 5.5 cells/mmc. The total sample size calculation required to enroll 60 patients in anticipation of a dropout rate of 15–20%. The reason for drop out of 11 patients was that at least one of their parents refused to consent to the child’s blood intake. Finally 49 patients of the Children’s Hospital of Lublin were included to this study (Table 1). The protocol included a total of three visits: screening/randomization visit (V1), biology visit (3 weeks; V2) and end-of-treatment visit (12 weeks; V3) [12].

**Table 1:** Demographic characteristics of tested population

Feature	PMBL® tablet	Placebo	*p*-value in Student t-test
Age (years) mean ± SD	10.67 ± 2.13	9.54 ± 2.52	0.44
Sex n (%)			
Male	21 (75)	18 (86)	0.34
Female	7 (25)	3 (14)	
Time since diagnosis of asthma (years) mean ± SD	4.51 ± 3.14	4.89 ± 3.23	0.87

### Complete blood count

At baseline, no significant differences between groups relative to blood count cells were observed, except for eosinophils. The eosinophils were 0.65 ± 0.39 G/L and 0.41 ± 0.26 G/L in PMBL® and Placebo groups respectively, p = 0.025. The median values for both groups indicate that at least 50% of the patients had high levels of eosinophils. Eosinophils represented 9.8 ± 5.9 versus 6.5 ± 3.8% of total white blood cells in PMBL® and Placebo groups, respectively.

The evolution of absolute values of red blood cells, total white blood cells, and platelets between baseline and 12 weeks was not significantly different in both groups of treatment (Table 2). In addition, absolute value of neutrophils, eosinophils, monocytes as well as basophils and their percent of white blood cells were similar at the baseline and at 12 weeks in both groups.

**Table 2:** Evolution of blood cells count values between baseline and 12 weeks (G/L)

Blood cells	Evolution between baseline and 12 weeks		p*
	PMBL® tablet N = 21 Mean ± SD	Placebo N = 28 Mean ± SD	
Total red blood cells	− 0.02 ± 0.27	+ 0.06 ± 0.29	0.4178
Total white blood cells	+ 0.4 ± 1.51	+ 0.13 ± 3.53	0.9046
Neutrophils (% WBC)	+ 0.27 ± 1.28 (+ 1.1 ± 9.6)	+ 0.33 ± 2.53 (+ 3.9 ± 9.7)	0.4697 (0.1727)
Basophils (% WBC)	− 0.00 ± 0.02 (− 0.06 ± 0.24)	0.00 ± 0.01 (+ 0.01 ± 0.29)	0.9838 (0.6610)
Eosinophils (% WBC)	+ 0.01 ± 0.21 (− 0.3 ± 3.6)	+ 0.06 ± 0.27 (0.5 ± 3.7)	0.8243 (0.8507)
Lymphocytes (% WBC)	+ 0.12 ± 0.49 (− − 0.2 ± 7.6)	− 0.30 ± 0.86 (− 5.2 ± 8.5)	*0.0141* (*0.1339*)
Monocytes (% WBC)	+ 0.01 ± 0.21 (− 0.7 ± 2.3)	+ 0.04 ± 0.57 (0.7 ± 2.5)	0.5564 (0.5102)
Platelet count	+ 0.7 ± 38.2	− 13.2 ± 45.3	0.2621

Evolution of lymphocytes as % WBC was significantly different between groups (in italics)

^*^ *p*-value based on the application of ANCOVA on ranks

Despite our recording of an increase percent of lymphocytes in the PMBL® group, when lymphocytes were compared with baseline (+ 0.2 ± 0.49 versus − 0.30 ± 0.86 in the Placebo group, p = 0.0141), the amount of T cells in the two groups did not differ at 12 weeks. Furthermore, while some individual laboratory data were out of normal ranges, none were considered as clinically significant and none was reported as treatment emergent adverse events (TEAE) by the investigators that were consistent with the allergic asthmatic status of the study population.

### White blood cells phenotypes

The baseline values of white blood cells phenotypes were comparable in both groups, whatever the way of description (percent or absolute count) except in the case of CD4+CD25+FOXP3+. The absolute values of lymphocytes expressing a Treg-like phenotype were 55 ± 22.7 and 71.8 ± 28.9 G/L in the PMBL® and Placebo groups, respectively (p = 0.039).

### *T lymphocytes (CD3*+*)*

The evolution of CD3+ absolute count was significantly different between groups at 12 weeks (p = 0.0165). Actually, the absolute count of T CD3+ cells increased in PMBL® group, but decreased in the Placebo group (Table 3). The observed changes should be considered with caution, considering the individual variability. The evolution of T CD3+ as percent of lymphocytes CD45+ was not significantly different between baseline and 12 weeks in both groups (p = 0.7031). Late activated T lymphocyte (CD3+CD25+) and early activated T lymphocytes (CD3+CD69+) evolution as percent of T CD3+ lymphocytes was significantly different between groups (p = 0.0243). A reduction was observed in the treated group, while in the Placebo group, an increase was detected.

**Table 3:** Evolution of lymphocytes (absolute count, G/L) between baseline and 12 weeks in the PMBL® and placebo groups

Subpopulation of lymphocytes	Evolution between baseline and 12 weeks		p*
	PMBL® Tablet N = 21 Mean ± SD	Placebo N = 28 Mean ± SD	
CD45+	+ 253.9 ± 750.7	− 289.7 ± 855.2	*0.0129*
CD3+	+ 240.9 ± 569.1	− 150.5 ± 471.0	*0.0165*
CD3+CD25+	+ 29.5 ± 149.9	− 21.3 ± 179.8	0.7955
CD3+CD25+ as % of CD3+	− 0.6 ± 5.3	+ 0.7 ± 4.1	*0.0243*
CD3+CD69+	− 6.2 ± 137.0	0.0 ± 259.9	0.0876
CD3+CD69+ as % of CD3+	− 2.2 ± 5.2	+ 2.5 ± 15.3	*0.0140*
CD3+CD4+	+ 113.8 ± 254.5	− 32.8 ± 214.8	0.0921
CD4+CD25+	+ 31.6 ± 119.9	− 19.0 ± 150.9	0.5996
CD4+CD25+ high	+ 3.6 ± 34.4	− 10.8 ± 37.0	0.8402
CD4+CD69+	− 3.3 ± 62.6	− 14.9 ± 110.9	0.6202
CD4+CD25+FOXP3+	+ 6.5 ± 37.3	− 10.8 ± 24.8	*0.0395*
CD3+CD8+	+ 136.6 ± 324.3	− 92.7 ± 282.8	*0.0181*
CD8+CD25+	− 28.2 ± 108.4	− 14.9 ± 95.5	0.9206
CD8+CD69+	− 13.6 ± 84.0	− 0.5 ± 43.7	0.9324
CD3+CD16+CD56+	+ 3.1 ± 44.9	− 4.9 ± 24.1	0.2760
CD3−CD16+CD56+	+ 19.1 ± 114.8	− 35.0 ± 164.0	*0.0463*

Evolution of CD45+, CD3+, CD3+CD25+ as % of CD3+, CD3+CD69+ as % of CD3+, CD4+CD25+FOXP3+, CD3+CD8+, CD3−CD16+CD56+ lymphocytes was significantly different between groups (in italics)

^*^ p-value based on application of ANCOVA on ranks

### T lymphocyte subpopulations

The evolution of CD3+CD4+ absolute count was not significantly different between groups, but approached significance (p = 0.0921). An increase was observed in treated patients, while a reduction was detected in the Placebo patients. The difference of CD3+CD4+ value as percent of CD45+ lymphocytes was not significant between the groups (p = 0.1808). The early and late activated T helper lymphocytes value evolution also did not differ between groups.

The evolution of CD3+CD8+ count significantly increased in treated patients and decreased in the Placebo group (p = 0.0181). The evolution of the ratio CD4/CD8 was not significantly different between Placebo and PMBL® groups (p = 0.387). The number of lymphocytes expressing a Treg phenotype (CD4+CD25+FOXP3) level significantly increased in the PMBL® group, while a reduction of Treg cells was observed in the Placebo group (p = 0.0395).

### NK cells

The evolution of NK cells (CD3-CD16+CD56+) absolute count was significantly different between groups (p = 0.0463). An increase was observed in PMBL® treated patients, while a reduction was detected in the Placebo group.

### Specific immunoglobulins

No difference was noted between groups whatever the concentration of PMBL® soluble antigen preparation used. Based on the baseline levels detected, all patients appeared to be immunocompetent as reflected by the polyclonal activity of PMBL® against multiple pathogens.

### Safety

Four serious adverse events (1 in the PMBL group and 3 in the placebo group) were reported during the study: 1 case in the PMBL group (paralysis of peripheral facial nerve) and 3 cases in the placebo group that led to hospitalization (acute laryngitis, asthma exacerbation, influenza). The investigator considered that none was related to study product intake. No serious adverse events related to PMBL® intake were reported, however, 3 patients reported 2 adverse events related to PMBL® intake: drowsiness and drug taste intolerance.

## Discussion

Chronic inflammation in airway is the central feature of asthma pathogenesis and the core of its clinical manifestations. If allergen exposure is not terminated or if appropriate treatment is not administered, chronically activated memory T cells with an enhanced capacity to produce IL-4 and IFN-γ, changed Th1/Th2 balance and changed of CD4^+^/CD8^+^ ratio in peripheral blood lymphocytes and in bronchial mucosa. It plays a key role in the pathophysiology that lead to structural changes in the airways, fixed airflow limitation and possibly greater hyperresponsiveness [22, 23]. Recurrent asthma exacerbations decrease quality of life of the patients by limiting their normal daily activities and by causing frequent hospital admissions [24].

The EOLIA study demonstrated that PMBL® administration 10 days per month for three months changed the panel of T lymphocytes. After 12 weeks of taking PMBL, we observed an increase of T lymphocytes (CD3+) count. This increase concerned Treg (CD4+CD25+FOXP3+), Tc (CD8+) and NK (CD3−CD16+CD56+), also, Th count insignificantly increased. The significant decrease of early activated (CD69+) and late activated (CD25+) subset of CD3+ could indicate a decrease of the chronic immune activation present in allergic asthmatic patients [25, 26].

Such a decrease of activation could account for the beneficial effect of PMBL® treatment on the clinical conditions of asthmatic children [24].

The increase of Treg (CD4+CD25+FOXP3+) after PMBL® treatment may be the possible mechanism that prevents asthma exacerbations and reduces the severity of this disease [13, 26]. The mechanism of Treg action is complex and multidirectional. Treg by direct contact suppresses proliferation and maturation and enhances apoptosis of the Th2 subpopulation of lymphocytes (T helper cell type 2). In this effect, IL-10, TGF-β and the cell surface molecules (CTLA-4, Notch-3 and LAG-3) are involved. It could be said, then that the production of pro-inflammatory cytokines (Il-4, 5, 13) is inhibited, while the eosinophils that prevent the development of airway remodeling, reduce mucus hypersecretion and peribronchial collagen deposition are inactivated [24, 27]. For this role, Treg must be still activated by allergen, bacteria or viruses. This fact is the basis of the hygiene hypothesis of allergy development and treatment by immunotherapy. The induction of tolerance may be obtained by breast-milk offspring feeding that delivers natural antigen, but also by allergen (specific immunotherapy) or inactivated pathogens (bacterial lysates) administration [27]. The data suggest that bacterial lysates decrease the symptoms of allergic disease and improve the comfort of life of allergic patients [18, 28, 29].

Most of the studies concern the oral administration of chemical lysate, OM-85, but the data about sublingual administration of mechanical lysate for human are limited. In our previous study, we described the clinical improvement of asthma treatment effects after using PMBL [12]. We suggest that the observed clinical effect could be dependent on immunological changes caused by PMBL, as the role of lymphocytes in atopic, as well as non-atopic asthma, is proven [24, 27, 30]. The longtime administration of bacterial lysates simulates the persistent present of low numbers of pathogens that chronically elevates levels of Treg cells activity. This nonspecific effect prevents the development of untoward allergic and autoimmune reactions via the mechanism deemed ‘bystander suppression [24, 31].

PMBL has increased the number of T and NK cells in patients with chronic obstructive pulmonary disease [5, 7]. We have not found similar studies in patients with asthma. In our study, the count of Th cells (CD3+CD4+) insignificantly increased. We did not divide Th cells into subpopulations, for that matter there are Th1 cells that have anti-viral and anti-microbial properties in this group, but also Th2 cells that are responsible for production pro-inflammatory cytokines activating eosinophils and allergic reaction [32].

The count of Tc cells (CD8+) in the treated group increased in our study, this suggests the stimulating impact of PMBL on immune response. The increase of Tc and NK cells level after PMBL® administration may suggest that decrease of asthma exacerbation in children is also dependent on a more effective response against viruses and other microbes responsible for respiratory infections, but this aspect need more studies.

In the Placebo group the count of CD3+, CD45+ and CD3+CD4+ decreased, that may be related to more frequent infections that have led to asthma exacerbations in these patients (described in our previous study) [12]. Similar correlation between proportion of paediatric patients with decreased CD3+, CD4+ and CD8+ T cells and severity of asthma exacerbation have shown Nguyen-Thi-Dieu et al. Author of this study observed that decrease of CD3+, CD4+, CD8+ T cells were associated with acute asthma exacerbation in children with asthma [33].

The increase in NK cells is most likely associated to a maturative effect of PMBL on dendritic cells whose ability of increasing the proliferation of NK cells upon bacterial stimuli is well documented [34]. Nevertheless, the increase in NK cells observed upon PMBL treatment might have helped in controlling viral infections, and, therefore, on reducing asthma exacerbations related to these infections [14]. The multidirectional effect of PMBL® on immunological parameters suggests that this lysate may have a preventive effect on both infection-related and -unrelated episodes of asthma exacerbation. Indeed, the therapeutic paradigm might be enhanced by increasing the duration of the treatment in the pathological context of allergic asthma.

Our results indicated the lack of stimulation of specific antibodies secretion by PMBL® in peripheral blood. No treatment-associated emergent adverse event has been reported. To our knowledge, this is the first time that a beneficial effect of PMBL as add-on therapy in allergic pediatric asthma treatment has been reported. However, a longer follow up, more numerous population and more detailed studies are needed to explain the mechanism of PMBL® action and its role in preventing asthma development and asthma exacerbation.

## Conclusions

A lyophilized Polyvalent Mechanical Bacterial Lysate administration in asthmatic children led to an increase of total T lymphocytes (CD3+) count. This increase concerned Treg (CD4+CD25+FOXP3+), Tc (CD8+) and NK (CD3−CD16+CD56+). The Th count was also, albeit, insignificantly increased. Meanwhile, the decrease of the early activated (CD69+) and late activated (CD25+) subset of CD3+ was significant after PMBL use. These changes in the panel of T lymphocytes could account for the beneficial effect of PMBL® treatment on the clinical conditions of asthmatic children relevant to respiratory system infections and asthma exacerbation, but this aspect needs more profound future studies.

## Data Availability

The datasets used and analysed during the current study are available from the corresponding author on reasonable request.

## References

[1] Morandi B, Agazzi A, D’Agostino A (2011). A mixture of bacterial mechanical lysates is more efficient than single strain lysate and of bacterial-derived soluble products for the induction of an activating phenotype in human dendritic cells. Immunol Lett.

[2] Jurkiewicz D, Zielnik-Jurkiewicz B (2018). Bacterial lysates in the prevention of respiratory tract infections. Otolaryngol Pol.

[3] Yin J, Xu B, Zeng X, Shen K (2018). Broncho-Vaxom in pediatric recurrent respiratory tract infections: A systematic review and meta-analysis. Int Immunopharmacol.

[4] Del-Rio-Navarro BE, Espinosa Rosales F, Flenady V, Sienra-Monge JJL (2006). Immunostimulants for preventing respiratory tract infection in children. Cochrane Database Syst Rev.

[5] Lanzilli G, Traggiai E, Braido F (2013). Administration of a polyvalent mechanical bacterial lysate to elderly patients with COPD: Effects on circulating T, B and NK cells. Immunol Lett.

[6] Braido F, Schenone G, Pallestrini E (2011). The relationship between mucosal immuno-response and clinical outcome in patients with recurrent upper respiratory tract infections treated with a mechanical bacterial lysate. J Biol Regul Homeost Agents.

[7] Lanzilli G, Falchetti R, Cottarelli A, Macchi A, Ungheri D, Fuggetta MP (2006). In vivo effect of an immunostimulating bacterial lysate on human B lymphocytes. Int J Immunopathol Pharmacol.

[8] Ricci R, Palmero C, Bazurro G (2014). The administration of a polyvalent mechanical bacterial lysate in elderly patients with COPD results in serological signs of an efficient immune response associated with a reduced number of acute episodes. Pulm Pharmacol Ther.

[9] Janeczek KP, Emeryk A, Rapiejko P (2019). Effect of polyvalent bacterial lysate on the clinical course of pollen allergic rhinitis in children. Postepy Dermatol Alergol.

[10] Janeczek KP, Emeryk A, Rapiejko P (2019). Effect of polyvalent bacterial lysate on the clinical course of pollen allergic rhinitis in children. Adv Dermatol Allergol Dermatol Alergol.

[11] Razi CH, Harmancı K, Abacı A (2010). The immuno-stimulant OM-85 BV prevents wheezing attacks in preschool children. J Allergy Clin Immunol.

[12] Emeryk A, Bartkowiak-Emeryk M, Raus Z, Braido F, Ferlazzo G, Melioli G (2018). Mechanical bacterial lysate administration prevents exacerbation in allergic asthmatic children-The EOLIA study. Pediatr Allergy Immunol.

[13] Lau S, Gerhold K, Zimmermann K (2012). Oral application of bacterial lysate in infancy decreases the risk of atopic dermatitis in children with 1 atopic parent in a randomized, placebo-controlled trial. J Allergy Clin Immunol.

[14] Castillo JR, Peters SP, Busse WW (2017). Asthma exacerbations: pathogenesis, prevention, and treatment. J Allergy Clin Immunol Pract.

[15] Busse WW, Lemanske RF, Gern JE (2010). Role of viral respiratory infections in asthma and asthma exacerbations. Lancet Lond Engl.

[16] Jackson DJ, Sykes A, Mallia P, Johnston SL (2011). Asthma exacerbations: origin, effect, and prevention. J Allergy Clin Immunol.

[17] Olenec JP, Kim WK, Lee W-M (2010). Weekly monitoring of children with asthma for infections and illness during common cold seasons. J Allergy Clin Immunol.

[18] Esposito S, Soto-Martinez ME, Feleszko W, Jones MH, Shen K-L, Schaad UB (2018). Nonspecific immuno-modulators for recurrent respiratory tract infections, wheezing and asthma in children: a systematic review of mechanistic and clinical evidence. Curr Opin Allergy Clin Immunol.

[19] Schatz M, Sorkness CA, Li JT (2006). Asthma Control Test: reliability, validity, and responsiveness in patients not previously followed by asthma specialists. J Allergy Clin Immunol.

[20] 2012-GINA.pdf. https://ginasthma.org/wp-content/uploads/2019/01/2012-GINA.pdf. Accessed 1 July 2019.

[21] Niu H, Wang R, Jia YT, Cai Y (2019). Pidotimod, an immunostimulant in pediatric recurrent respiratory tract infections: a meta-analysis of randomized controlled trials. Int Immunopharmacol.

[22] Abdulamir AS, Hafidh RR, Abubakar F, Abbas KA (2008). Changing survival, memory cell compartment, and T-helper balance of lymphocytes between severe and mild asthma. BMC Immunol.

[23] Waserman S, Nair P, Snider D (2012). Local and systemic immunological parameters associated with remission of asthma symptoms in children. Allergy Asthma Clin Immunol..

[24] Branchett WJ, Lloyd CM (2019). Regulatory cytokine function in the respiratory tract. Mucosal Immunol.

[25] Bajnok A, Ivanova M, Rigó J, Toldi G (2017). The distribution of activation markers and selectins on peripheral T lymphocytes in preeclampsia. Mediators Inflamm..

[26] Cibrián D, Sánchez-Madrid F (2017). CD69: from activation marker to metabolic gatekeeper. Eur J Immunol.

[27] Ryanna K, Stratigou V, Safinia N, Hawrylowicz C (2009). Regulatory T cells in bronchial asthma. Allergy.

[28] de Boer GM, Braunstahl G-J, Hendriks RW, Tramper GA (2018). Bacterial lysates in the prevention of asthma exacerbations in uncontrolled asthma: the Breathe study. Eur Respir J..

[29] Han R-F, Li H-Y, Wang J-W, Cong X-J (2016). Study on clinical effect and immunologic mechanism of infants capillary bronchitis secondary bronchial asthma treated with bacterial lysates Broncho-Vaxom. Eur Rev Med Pharmacol Sci.

[30] Salter BM, Aw M, Sehmi R (2019). The role of type 2 innate lymphoid cells in eosinophilic asthma. J Leukoc Biol.

[31] Navarro S, Cossalter G, Chiavaroli C (2011). The oral administration of bacterial extracts prevents asthma via the recruitment of regulatory T cells to the airways. Mucosal Immunol.

[32] Gołąb J, Jakóbisiak M, Lasek W, Stokłosa T, ed. Immunologia. PWN; 2017.

[33] Nguyen-Thi-Dieu T, Le-Thi-Thu H, Duong-Quy S (2017). The profile of leucocytes, CD3+, CD4+, and CD8+ T cells, and cytokine concentrations in peripheral blood of children with acute asthma exacerbation. J Int Med Res.

[34] Walwyn-Brown K, Guldevall K, Saeed M (2018). Human NK cells Lyse Th2-polarizing dendritic cells via NKp30 and DNAM-1. J Immunol.

